# Prognostic Value of the Neutrophil Percentage-to-Albumin Ratio in Acute Non-Variceal Upper Gastrointestinal Bleeding

**DOI:** 10.3390/jcm15082854

**Published:** 2026-04-09

**Authors:** Ahmet Yavuz, Ümit Karabulut, Berat Ebik, Mustafa Zanyar Akkuzu, Ferhat Bingöl

**Affiliations:** 1 Department of Gastroenterology, Diyarbakır Gazi Yasargil Education and Research Hospital, University of Health Sciences, Diyarbakır 21070, Turkey; drumitkarabulut@gmail.com (Ü.K.); beratebik@gmail.com (B.E.); zanyarakkuzu@gmail.com (M.Z.A.); 2 Department of Internal Medicine, Diyarbakır Gazi Yasargil Education and Research Hospital, University of Health Sciences, Diyarbakır 21070, Turkey; fbingol2184@gmail.com

**Keywords:** non-variceal upper gastrointestinal bleeding (NVUGIB), inflammation-based indices, neutrophil percentage-to-albumin ratio (NPAR), AIMS65, Rockall score

## Abstract

**Background:** Early risk assessment in non-variceal upper gastrointestinal bleeding (NVUGIB) is essential for guiding clinical management. The neutrophil percentage-to-albumin ratio (NPAR) has recently been proposed as a marker reflecting both inflammatory response and physiological reserve. This study aimed to evaluate the prognostic value of NPAR for in-hospital mortality and its relationship with established risk scores in patients with NVUGIB. **Methods:** This retrospective observational study included 94 patients hospitalized with NVUGIB. NPAR was calculated using laboratory parameters obtained at admission. Patients were stratified according to AIMS65 (<2 vs. ≥2) and Rockall (<5 vs. ≥5) scores. In addition, inflammation-based indices, including neutrophil-to-lymphocyte ratio (NLR), platelet-to-lymphocyte ratio (PLR), and systemic immune-inflammation index (SII), were calculated. Predictive performance was evaluated using receiver operating characteristic (ROC) curve analysis, and associations with clinical outcomes were assessed. **Results:** The in-hospital mortality rate was 12.8%. NPAR values were significantly higher in patients with AIMS65 ≥ 2 and Rockall ≥ 5 (*p* < 0.001 for both). NPAR demonstrated good discriminative ability for AIMS65 ≥ 2 (AUC: 0.843) and moderate performance for Rockall ≥ 5 (AUC: 0.714). For mortality prediction, NPAR showed excellent performance (AUC: 0.900). A cut-off value of 27.4 yielded a sensitivity of 91.7% and a specificity of 75.6%. Higher NPAR values were associated with increased mortality risk (OR 31.9, 95% CI: 3.88–102.59, *p* < 0.001), while the negative predictive value was high (98.4%). In contrast, NLR, PLR, and SII showed limited predictive value for in-hospital mortality. **Conclusions:** NPAR shows promise as a potential prognostic biomarker for assessing disease severity and in-hospital mortality in NVUGIB. Its high negative predictive value and association with established risk scores suggest that it may complement current risk stratification approaches. However, these findings should be considered preliminary, given the retrospective design and limited sample size, and require validation in larger prospective studies.

## 1. Introduction

Non-variceal upper gastrointestinal bleeding (NVUGIB) is defined as blood loss originating from the gastrointestinal tract proximal to the ligament of Treitz (the duodenojejunal junction). The reported annual incidence ranges from 15 to 172 per 100,000 individuals [1]. Despite advances in diagnostic and therapeutic modalities, NVUGIB remains a major cause of morbidity and mortality, with mortality rates reported between 3% and 14% [2,3,4]. Advanced age, hemodynamic instability, and the presence of comorbid conditions are well-established factors that significantly increase the risk of death [5]. Therefore, early prediction of mortality risk in patients with NVUGIB is of critical importance for clinical decision-making.

Several risk stratification systems, including the Forrest classification, Glasgow–Blatchford score (GBS), Rockall score, and AIMS65, have been developed and are widely used in clinical practice for patients with NVUGIB [6,7,8,9]. These scores provide valuable prognostic information through their multivariable structure, incorporating age, vital signs, laboratory parameters, and comorbidities. However, the requirement for endoscopic findings in some scoring systems and the inclusion of multiple variables may limit their early and practical use in emergency settings. This limitation has driven growing interest toward simple, rapidly obtainable prognostic markers based on routine laboratory parameters.

The impact of systemic inflammation on the clinical course and prognosis of NVUGIB has gained growing attention. Several studies have demonstrated that inflammatory markers such as the platelet-to-lymphocyte ratio (PLR), neutrophil-to-lymphocyte ratio (NLR), and systemic immune-inflammation index (SII) are associated with prolonged hospital stay and increased mortality in NVUGIB [10,11,12]. While the neutrophil percentage is considered a marker of the inflammatory response, serum albumin level is widely used as an indirect indicator of nutritional status and systemic stress. The neutrophil percentage-to-albumin ratio (NPAR) is a novel index that combines these two parameters and simultaneously reflects inflammatory burden and physiological reserve [13]. NPAR has been reported to be associated with adverse outcomes in various clinical conditions, including sepsis, pneumonia, cardiovascular diseases, malignancies, and variceal bleeding [14,15,16,17]. However, to the best of our knowledge, the prognostic value of NPAR in patients with NVUGIB has not been previously investigated.

The aim of this study was to evaluate the prognostic value of the NPAR in patients with NVUGIB, to investigate its association with established clinical risk scores (AIMS65 and Rockall), and to compare its predictive performance with other inflammation-based biomarkers, including NLR, PLR, and SII, in predicting in-hospital mortality.

## 2. Materials and Methods

### 2.1. Study Design and Population

This study was designed as a retrospective observational analysis of patients managed for NVUGIB in the gastroenterology department of our institution between January 2025 and January 2026. Ethical approval was obtained from the local ethics committee of the University of Health Sciences Gazi Yaşargil Training and Research Hospital (January 2026; approval no: 14), and the study adhered to the principles outlined in the Declaration of Helsinki.

Adult patients (≥18 years) who were hospitalized due to NVUGIB were eligible for inclusion. Exclusion criteria comprised variceal bleeding, bleeding related to upper gastrointestinal malignancies, extra-gastrointestinal malignancies, acute myocardial infarction, acute cerebrovascular events, pulmonary embolism, chronic inflammatory disorders, pregnancy, ongoing immunosuppressive therapy, and prior albumin replacement. Patients with evidence of acute infection at admission were also excluded due to their potential influence on neutrophil parameters. Furthermore, patients managed in the outpatient setting, those lacking albumin measurements at admission, and those with incomplete data required for AIMS65 or Rockall score calculation were excluded.

### 2.2. Data Collection

Baseline demographic data (age and sex), comorbid conditions (hypertension, diabetes mellitus, chronic kidney disease, coronary artery disease, and cerebrovascular disease) and medication history (anticoagulants, antiplatelet agents, and non-steroidal anti-inflammatory drugs) were recorded. Due to the limited sample size, comorbidity burden was evaluated as a binary variable (presence versus absence of at least one chronic condition).

Clinical variables, including blood pressure, syncope at presentation, etiology of bleeding, transfusion requirements, length of hospital stay, and rebleeding events, were also documented. Hypotension was defined as a mean arterial pressure below 60 mmHg.

Laboratory parameters obtained at admission included hemoglobin, hematocrit, neutrophil count, neutrophil percentage, urea, creatinine, and albumin levels. According to institutional protocol, blood samples were routinely obtained at the time of initial intravenous access in the emergency department, prior to the initiation of fluid resuscitation. All-cause in-hospital mortality was recorded as the primary outcome.

### 2.3. Patient Management and Endoscopic Evaluation

All patients received proton pump inhibitor (PPI) infusion and intravenous saline or Ringer’s lactate for hemodynamic stabilization in accordance with current guideline recommendations. Erythrocyte suspension transfusion was performed to maintain hemoglobin levels between 7 and 9 g/dL in hemodynamically stable patients without cardiovascular disease and ≥10 g/dL in patients with cardiovascular disease [18].

Upper gastrointestinal endoscopy was performed within the first 24 h after clinical stabilization. Peptic ulcer bleeding was classified according to the Forrest classification [19]. Endoscopic hemostatic therapy was performed in high-risk lesions (Forrest 1a, 1b, 2a, and 2b) [20]. In patients in whom hemostasis could not be achieved endoscopically, transcatheter arterial embolization (TAE) or surgical intervention was performed according to the patient’s clinical condition. For statistical analysis, endoscopic findings were dichotomized into high-risk (Forrest I–IIb) and low-risk (Forrest IIc–III) groups. Patients with non-peptic bleeding etiologies were excluded from analyses involving Forrest classification. Antiplatelet and anticoagulant therapies were continued or discontinued according to the relevant clinical decisions.

### 2.4. Risk Scores and Inflammation-Based Indices

For each patient, the AIMS65 score (albumin, international normalized ratio (INR), mental status, blood pressure, and age) was calculated at hospital admission, and the Rockall score (age, comorbidity, hemodynamic status, diagnosis, and stigmata of recent hemorrhage) was calculated after upper gastrointestinal endoscopy.

The study population was stratified into low- and high-risk groups according to the established cut-off values of the AIMS65 score (<2 vs. ≥2) and the Rockall score (<5 vs. ≥5).

NPAR was calculated using laboratory data obtained at admission as follows: neutrophil percentage (%)/albumin (g/dL).

In addition, NLR, PLR, and SII were calculated using admission laboratory values. NLR was defined as the ratio of neutrophil count to lymphocyte count, PLR as the ratio of platelet count to lymphocyte count, and SII as platelet count × neutrophil count/lymphocyte count.

The relationships between NPAR, other inflammatory biomarkers (NLR, PLR, and SII), clinical risk scores (AIMS65 and Rockall), and in-hospital outcomes were evaluated.

### 2.5. Statistical Analysis

All statistical analyses were conducted using SPSS version 24.0 (IBM Corp., Armonk, NY, USA). Data distribution was evaluated using Kolmogorov–Smirnov and Shapiro–Wilk tests, along with assessment of skewness and kurtosis. Continuous variables were expressed as mean ± standard deviation when normally distributed, and as median (interquartile range) when non-normally distributed. Categorical variables were presented as frequencies and percentages.

Group comparisons were performed using the independent samples t-test or Mann–Whitney U test, depending on data distribution. Categorical variables were analyzed using the chi-square or Fisher’s exact test, as appropriate.

Associations between NPAR and clinical parameters, including endoscopic risk classification, rebleeding, and comorbidity status, were evaluated using non-parametric methods.

Receiver operating characteristic (ROC) curve analysis was used to assess the discriminative performance of NPAR for predicting AIMS65 ≥ 2, Rockall ≥ 5, and in-hospital mortality. The area under the curve (AUC) was calculated with corresponding 95% confidence intervals. Sensitivity, specificity, and predictive values were determined for the identified thresholds. Additional ROC analyses were conducted for age, neutrophil percentage, albumin, and other inflammatory indices (NLR, PLR, and SII).

Correlation analyses were performed using Spearman’s rank correlation coefficient. A bivariable logistic regression model adjusted for age was constructed to evaluate the association between NPAR and mortality. Model calibration was assessed using the Hosmer–Lemeshow test, and explanatory power was evaluated using Cox & Snell and Nagelkerke R^2^ statistics.

A two-tailed *p* value < 0.05 was considered statistically significant.

## 3. Results

A total of 94 patients with NVUGIB were included in the study. The median age was 67 years (IQR: 50–79; range: 21–96), and 67.0% were male. A substantial proportion of patients had underlying comorbid conditions. The most frequent comorbidity was hypertension (39.4%), followed by coronary artery disease (28.7%), diabetes mellitus (21.3%), cerebrovascular disease (16.0%), and chronic kidney disease (9.6%). Anticoagulant use was present in 11.7% of patients, antiplatelet therapy in 21.3%, and nonsteroidal anti-inflammatory drug (NSAID) use in 31.9% of the cohort. Peptic ulcer was the most common etiology of bleeding (91.5%). Endoscopic hemostatic therapy was performed in 36 patients (38.3%), while 3 (3.2%) patients required TAE and 2 (2.1%) patients underwent surgical intervention. According to risk stratification scores, 40.4% of patients had an AIMS65 score ≥ 2, and 54.3% had a Rockall score ≥ 5. The median length of hospital stay was 3 days (IQR: 2–4; range: 1–18), and the median number of erythrocyte suspension transfusions was 3 units (IQR: 1–4; range: 0–17). Rebleeding occurred in 8.5% of patients. Overall, all-cause in-hospital mortality was 12.8% (Table 1).

Patients classified as high risk (AIMS65 ≥ 2) were significantly older than those with AIMS65 < 2, with median ages of 79.5 (73–86.2) and 53.0 (37.2–65), respectively (*p* < 0.001). Hemoglobin and albumin levels were significantly lower in the high-risk group (*p* = 0.007 and *p* < 0.001, respectively), whereas urea and creatinine levels were significantly higher (*p* = 0.007 and *p* < 0.001, respectively). Neutrophil percentage was also higher in patients with AIMS65 ≥ 2 (*p* = 0.030). The median length of hospital stay was longer in patients with AIMS65 ≥ 2 compared to those with AIMS65 < 2, 4 (3–5) vs. 2 (1–3) days (*p* < 0.001), and the number of erythrocyte suspension transfusions was significantly higher, 3 (2–5.2) vs. 1 (0–3) (*p* = 0.004). Although rebleeding was more frequent in the high-risk group, this difference did not reach statistical significance (15.78% vs. 3.57%, *p* = 0.058). NPAR values were significantly higher in patients with AIMS65 ≥ 2 compared to those with AIMS65 < 2, 30.5 (25.1–35.2) vs. 21.8 (18.2–25.1) (*p* < 0.001). Among other inflammation-based indices, NLR was also higher in the high-risk group (*p* = 0.034), whereas PLR and SII did not differ significantly between the groups (*p* = 0.089 and *p* = 0.343, respectively). In-hospital mortality was significantly higher in patients with AIMS65 ≥ 2 compared to those with AIMS65 < 2 (28.9% vs. 1.8%, *p* < 0.001) (Table 2).

Similarly, patients with a Rockall score ≥ 5 were significantly older than those with a score < 5, with median ages of 77 (65–83) and 51 (33–65), respectively (*p* < 0.001). Hemoglobin and albumin levels were significantly lower in the high-risk group (*p* = 0.001 and *p* < 0.001, respectively), whereas urea and creatinine levels were significantly higher (*p* = 0.048 and *p* < 0.001, respectively). The median length of hospital stay was longer in patients with Rockall ≥ 5 compared to those with Rockall < 5, 3 (2–5) vs. 2 (1–3) days (*p* = 0.001), and the number of erythrocyte suspension transfusions was significantly higher, 3 (2–5) vs. 1 (0–3) (*p* = 0.001). Rebleeding occurred only in the high-risk group (15.7% vs. 0%, *p* = 0.007). NPAR values were significantly higher in patients with Rockall ≥ 5 compared to those with Rockall < 5, 27.4 (21.8–33) vs. 21.9 (19.4–25.7) (*p* < 0.001). Among other inflammation-based indices, NLR, PLR, and SII did not differ significantly between the groups (*p* = 0.311, *p* = 0.260, and *p* = 0.663, respectively). In-hospital mortality occurred exclusively in the high-risk Rockall group (23.5% vs. 0%, *p* = 0.001) (Table 3).

**Figure 1:**
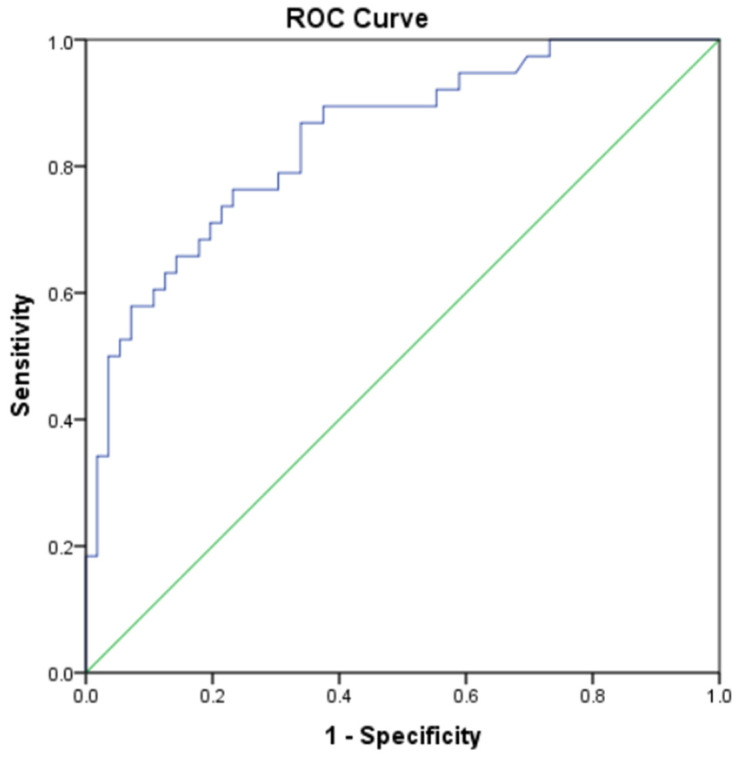
Receiver operating characteristic (ROC) curve of neutrophil percentage-to-albumin ratio (NPAR) for predicting an AIMS65 score ≥ 2. The blue line represents the ROC curve of NPAR, and the green diagonal line indicates the line of no discrimination (AUC = 0.5).

ROC analysis indicated that NPAR had good diagnostic performance for detecting AIMS65 ≥ 2 (AUC: 0.843, 95% CI: 0.763–0.923, *p* < 0.001). At a cut-off level of 23.5, sensitivity and specificity were 86.8% and 66.1%, respectively (Figure 1, Table 4).

**Figure 2:**
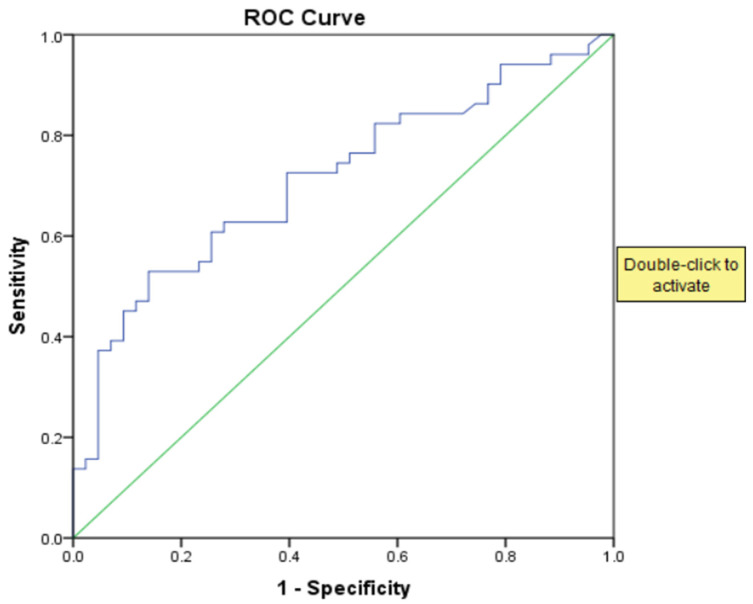
ROC curve of NPAR for predicting a Rockall score ≥ 5. The blue line represents the ROC curve of NPAR, while the green diagonal line represents the line of no discrimination (AUC = 0.5).

For the prediction of Rockall scores ≥ 5, NPAR demonstrated moderate discriminative performance (AUC: 0.714, 95% CI: 0.610–0.817, *p* < 0.001). A cut-off value of 21.2 corresponded to a sensitivity of 82.4% and a specificity of 45.0% (Figure 2, Table 4).

**Figure 3:**
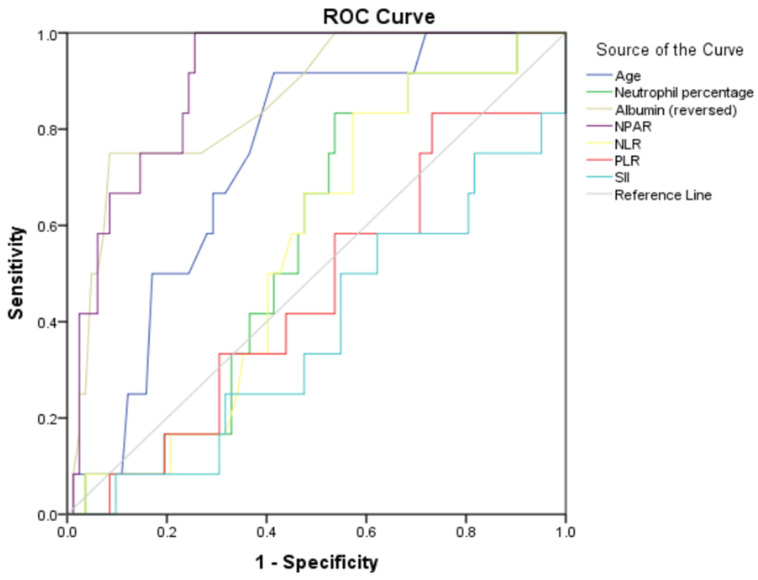
ROC curves of NPAR, albumin (reversed), neutrophil percentage, NLR, PLR, SII, and age for predicting in-hospital mortality. Albumin values were reversed for ROC analysis so that higher values indicate higher mortality risk.

Notably, NPAR exhibited excellent predictive value for in-hospital mortality (AUC: 0.900, 95% CI: 0.832–0.969, *p* < 0.001). An optimal cut-off value of 27.4 was identified, yielding a sensitivity of 91.7% and a specificity of 75.6%. Among the other parameters, albumin (reversed) also demonstrated good predictive performance (AUC: 0.860, 95% CI: 0.754–0.966, *p* < 0.001), while age showed moderate discriminative ability (AUC: 0.740, 95% CI: 0.618–0.862, *p* = 0.007). In contrast, neutrophil percentage, NLR, PLR, and SII did not show significant predictive value for in-hospital mortality (AUC: 0.562, *p* = 0.489; AUC: 0.552, *p* = 0.560; AUC: 0.454, *p* = 0.610; and AUC: 0.376, *p* = 0.167, respectively) (Figure 3, Table 4).

Patients with high-risk endoscopic findings had significantly higher NPAR values compared to those with low-risk lesions (*p* = 0.049). Although NPAR values were higher in patients with comorbidities, the difference did not reach statistical significance (*p* = 0.060). NPAR values were significantly higher in patients with rebleeding compared to those without rebleeding (median: 31.8 vs. 23.8, *p* = 0.004). In addition, patients requiring repeat endoscopy had significantly higher NPAR values compared to those who did not undergo repeat endoscopy (median: 30.6 vs. 23.8, *p* = 0.003).

Spearman correlation analysis demonstrated significant positive correlations between NPAR and several clinical parameters. NPAR showed a strong positive correlation with AIMS65 score (r = 0.660, *p* < 0.001) and hospitalization length (r = 0.636, *p* < 0.001). In addition, NPAR was moderately correlated with erythrocyte suspension transfusion requirements (r = 0.559, *p* < 0.001) and Rockall score (r = 0.491, *p* < 0.001) (Table 5).

In a bivariable logistic regression analysis adjusted for age, NPAR remained independently associated with in-hospital mortality (OR: 1.162, 95% CI: 1.065–1.268, *p* = 0.001), whereas age was not a significant predictor (*p* = 0.128). The logistic regression model demonstrated good calibration, as indicated by the Hosmer–Lemeshow goodness-of-fit test (χ^2^ = 5.421, *p* = 0.712). The model also showed acceptable explanatory power (Nagelkerke R^2^ = 0.418; Cox & Snell R^2^ = 0.223) (Table 6).

At a threshold of 27.4, elevated NPAR values were strongly associated with an increased risk of in-hospital mortality (OR: 31.9; 95% CI: 3.88–102.59; *p* < 0.001). The positive predictive value was 34.4%, while the negative predictive value reached 98.4%, highlighting the strong ability of low NPAR levels to exclude mortality (Table 7).

## 4. Discussion

In this study, NPAR was found to be significantly associated with established clinical risk scores and in-hospital mortality in patients with NVUGIB. Higher NPAR values were observed in patients with elevated AIMS65 and Rockall scores, and NPAR demonstrated good discriminative performance for both disease severity and mortality outcomes. These findings suggest that NPAR may represent a practical and biologically relevant parameter for early risk assessment in NVUGIB.

Risk stratification is a cornerstone in the management of NVUGIB, and several scoring systems have been developed for this purpose. The Rockall score, one of the earliest validated tools, was designed to predict mortality and rebleeding by incorporating both clinical and endoscopic parameters [8]. In contrast, the AIMS65 score was introduced as a simplified bedside model that predicts in-hospital mortality using readily available clinical and laboratory variables without the need for endoscopic findings [9]. In our study, both AIMS65 and Rockall high-risk groups were characterized by significantly higher transfusion requirements, longer hospital stays, increased rebleeding rates, and higher in-hospital mortality. This finding is consistent with the literature and confirms that these scoring systems not only reflect theoretical risk but also correlate with clinically relevant outcomes [21,22]. The higher need for blood transfusion in the high-risk groups indicates more severe bleeding and greater hemodynamic compromise, while the increased mortality further supports their established prognostic value.

We found no significant difference in leukocyte and neutrophil counts between high- and low-risk groups according to AIMS65 and Rockall scores. Although leukocytosis may occur in patients with NVUGIB, studies investigating its clinical significance are limited [23,24,25]. Chalasani et al. [23] reported leukocyte counts >20,000/mm^3^ in more than 5% of patients and suggested that leukocytosis might reflect bleeding severity and the amount of blood loss. However, no significant association with mortality was observed. Similarly, Srygley et al. demonstrated that hemoglobin < 8 g/dL, blood urea nitrogen >90 mg/dL, and leukocyte count >12,000/mm^3^ were associated with increased bleeding severity [24]. Kandemir et al. [25] detected leukocytosis in 48.7% of NVUGIB patients but did not find a significant relationship between leukocytosis and bleeding severity or mortality. These findings suggest that leukocyte count alone may have limited value in risk stratification.

In our cohort, albumin levels were significantly lower in patients with high AIMS65 and Rockall scores. Hypoalbuminemia has been consistently reported as a strong prognostic factor in NVUGIB. Previous studies have shown that low serum albumin levels are associated with increased bleeding severity, higher transfusion requirements, prolonged hospital stay, rebleeding, and increased in-hospital mortality [26,27,28]. Notably, the predictive performance of serum albumin for mortality has been reported to be comparable to that of the Rockall score, emphasizing that baseline physiological reserve rather than the bleeding lesion alone plays a central role in determining clinical outcomes in NVUGIB [27].

We also demonstrated that NPAR values were significantly higher in patients with high AIMS65 and Rockall scores and in those who developed in-hospital mortality. In recent years, the prognostic value of NPAR has been reported in various clinical conditions. Elevated NPAR has been associated with poor functional outcomes in spontaneous intracerebral hemorrhage, increased mortality in atrial fibrillation, adverse prognosis in chronic heart failure, and worse clinical outcomes in liver cirrhosis [29,30,31,32]. These findings support the concept that NPAR carries a composite signal reflecting both inflammatory activation and reduced host reserve. Our study extends this concept to NVUGIB.

The pathophysiological rationale for utilizing NPAR is grounded in two complementary mechanisms. Neutrophilia reflects acute systemic inflammatory activation, whereas hypoalbuminemia represents chronic inflammatory burden, malnutrition, and reduced physiological reserve [33,34]. Albumin is not only a nutritional marker but also an antioxidant and anti-inflammatory molecule, and low levels have consistently been associated with increased morbidity and mortality in critically ill patients [35,36]. This mechanism may be even more pronounced in elderly patients, who constituted a substantial proportion of the high-risk groups in our cohort and in whom chronic low-grade inflammation and hypoalbuminemia are common [37]. Therefore, integrating inflammatory and nutritional status into a single ratio may provide incremental prognostic information beyond isolated laboratory parameters.

It is also important to consider the potential confounding effect of chronic liver disease on NPAR values. Although patients with variceal bleeding were excluded, individuals with compensated chronic liver disease may still have been included in the study cohort. In such patients, baseline hypoalbuminemia related to impaired hepatic synthetic function may lead to elevated NPAR values independent of the acute bleeding episode. Therefore, the observed association between NPAR and clinical outcomes may, in part, reflect underlying liver function rather than bleeding severity alone. This potential confounding effect should be taken into account when interpreting the results.

In addition to NPAR, we evaluated other inflammation-based indices, including NLR, PLR, and SII. Although these markers have demonstrated prognostic value in some previous studies, they did not show statistically significant predictive performance for in-hospital mortality in our cohort [10,11]. This discrepancy may be related to the relatively small sample size and limited number of events, which may have reduced statistical power. Moreover, indices based solely on blood cell counts may be less reflective of overall physiological status compared to NPAR, which incorporates albumin as a marker of systemic reserve.

In addition to mortality, we also evaluated several clinically relevant outcomes, including transfusion requirements, length of hospital stay, rebleeding, repeat endoscopy, endoscopic risk classification, and comorbidity burden. Higher NPAR values were associated with increased transfusion needs and longer hospitalization, suggesting a relationship with overall disease severity and clinical burden. NPAR was also significantly higher in patients with high-risk endoscopic findings according to the Forrest classification, in those who developed rebleeding, and in patients requiring repeat endoscopy, further supporting its potential role in identifying patients with more severe bleeding profiles. Although NPAR values tended to be higher in patients with comorbid conditions, this association did not reach statistical significance, which may be related to the limited sample size. In addition, older age was more frequently observed in high-risk groups, consistent with previous literature indicating age as an important determinant of outcomes in NVUGIB [5,6].

In our cohort, NPAR showed good predictive ability for identifying patients with AIMS65 ≥ 2 (AUC 0.843), moderate predictive ability for Rockall ≥ 5 (AUC 0.714), and excellent discrimination for in-hospital mortality (AUC 0.900). An NPAR cut-off value of 27.4 was identified for mortality prediction and was associated with a marked increase in the probability of death. While this threshold demonstrated strong diagnostic performance within our cohort, it should be interpreted as exploratory and hypothesis-generating given the retrospective design and limited sample size. To our knowledge, no previous study has evaluated the prognostic value of NPAR in NVUGIB. However, Mousa et al. [17] reported a very similar cut-off value (27.8) for predicting mortality in patients with variceal bleeding. This similarity suggests that NPAR may reflect a common pathophysiological pathway across different subgroups of upper gastrointestinal bleeding. Although the positive predictive value (34.4%) for mortality was modest, the strong negative predictive value (98.4%) highlights the potential role of NPAR as a rule-out biomarker. In high-volume emergency departments or resource-limited settings, such a parameter may support rapid triage decisions before full risk score calculation or endoscopic evaluation. Moreover, the combination of a low NPAR value with a low AIMS65 score may help identify patients at very low risk of in-hospital mortality, who could potentially be considered for early discharge or outpatient management. However, this approach should be interpreted cautiously and requires prospective validation.

Given the well-established influence of age on outcomes in NVUGIB, we performed a bivariable logistic regression analysis adjusted for age. Notably, NPAR remained significantly associated with in-hospital mortality after adjustment, indicating that its predictive value is not solely driven by age differences. However, due to the limited number of events, further multivariable analyses were not feasible and these findings should be interpreted with caution.

The in-hospital mortality rate observed in our study (12.8%) appears higher than that reported in large-scale studies of NVUGIB [3,4]. This may be explained by the inclusion of hospitalized patients only, as less severe cases are often managed in the outpatient setting and were not captured in our cohort. In addition, our center functions as a tertiary referral hospital, where patients with more severe clinical presentations and multiple comorbidities are more likely to be admitted. Furthermore, all-cause in-hospital mortality was evaluated, and deaths unrelated to bleeding may have been included. These factors may have contributed to the relatively higher mortality rate and should be considered when interpreting the predictive performance of NPAR.

This study has several limitations. First, the relatively small sample size and limited number of mortality events may have affected the stability of statistical estimates and precluded comprehensive multivariable analysis. Second, the retrospective, single-center design may limit the generalizability of the findings. Third, although laboratory parameters were obtained prior to fluid resuscitation, the potential impact of hemodilution on albumin levels and NPAR cannot be entirely excluded. In addition, only baseline NPAR values were evaluated, and dynamic changes over time were not assessed. The identified NPAR cut-off value was derived from the study cohort and should be considered exploratory, and hypothesis-generating, as it has not been externally validated. Furthermore, potential confounders such as chronic liver disease and the effects of hemodilution on albumin levels cannot be fully excluded. In addition, subgroup analyses according to bleeding etiology, age groups, and comorbidity burden could not be performed due to the limited sample size, which represents an additional limitation of the study. Finally, some clinically relevant variables, including detailed timing of endoscopy and certain procedural outcomes, were not systematically available due to the retrospective design.

Despite these limitations, our study also has important strengths. All laboratory parameters were obtained at admission, supporting the clinical applicability of NPAR in early decision-making. Moreover, NPAR was evaluated using multiple analytical approaches, including group comparisons, ROC analysis, correlation analysis, and odds ratio assessment, and showed consistent associations with severity and mortality endpoints.

## 5. Conclusions

In conclusion, NPAR shows promise as a potential prognostic biomarker for predicting disease severity and in-hospital mortality in patients with NVUGIB. Its strong negative predictive value and significant association with established risk scores suggest that it may complement current risk stratification tools. However, these findings should be considered preliminary, as they are derived from a relatively small, single-center, retrospective cohort. Further validation in larger, prospective, multicenter studies is required before NPAR can be incorporated into routine clinical practice. 

## Figures and Tables

**Table 1:** Demographic, clinical, and endoscopic characteristics of patients with upper gastrointestinal bleeding.

Parameter	*n* (%) or Value
Total number of patients	94 (100%)
Age, years	67 (50–79), range 21–96
**Sex**	
Female	31 (33.0)
Male	63 (67.0)
**Comorbidities**	
Hypertension	37 (39.4)
Diabetes mellitus	20 (21.3)
Chronic kidney disease	9 (9.6)
Coronary artery disease	27 (28.7)
Cerebrovascular disease	15 (16.0)
Anticoagulant use	11 (11.7)
Antiplatelet use	20 (21.3)
NSAID use	30 (31.9)
**Etiology of bleeding**	
Peptic ulcer	86 (91.5)
Esophageal ulcer	7 (7.4)
Mallory–Weiss tear	1 (1.1)
**Forrest classification**	
Ia	1 (1.1)
Ib	7 (8.0)
IIa	12 (13.8)
IIb	16 (18.4)
IIc	6 (6.9)
III	45 (51.7)
Syncope	8 (8.5)
Hypotension	32 (34.0)
**Rockall score**	
<5	43 (45.7)
≥5	51 (54.3)
**AIMS65 score**	
<2	56 (59.6)
≥2	38 (40.4)
Hospitalization length (day)	3 (2–4), range 1–18
Erythrocyte suspension transfusions, units	3 (1–4), range 0–17
Rebleeding	8 (8.5)
Repeat endoscopy	9 (9.5)
Mortality	12 (12.8)

Data are presented as number (percentage) or median (interquartile range, IQR) with range. NSAIDs: Non-steroidal anti-inflammatory drugs.

**Table 2:** Comparison of clinical and laboratory parameters according to AIMS65 score.

Parameter	AIMS65 < 2 (*n* = 56)	AIMS65 ≥ 2 (*n* = 38)	*p*-Value
Age, years	53 (37.25–65)	79.5 (73–86.2)	<0.001
Hemoglobin, g/dL	9.8 ± 2.8	8.2 ± 2.3	0.007
Platelet count, ×10^9^/L	269 (208–317)	236 (169–331)	0.234
White blood cell count, ×10^9^/L	9.9 ± 3.6	9.2 ± 3.8	0.401
Neutrophil count, ×10^9^/L	7.1 ± 3.5	7.1 ± 3.4	0.975
Neutrophil percentage, %	70 ± 12.1	75 ± 9.5	0.030
Albumin, g/dL	3.2 ± 0.4	2.5 ± 0.5	<0.001
Urea, mg/dL	65.5 (45–81)	90.5 (58–133)	0.007
Creatinine, mg/dL	0.78 (0.7–0.9)	1 (0.9–2.1)	<0.001
Hospitalization length (days)	2 (1–3)	4 (3–5)	<0.001
Erythrocyte suspension transfusions, units	1 (0–3)	3 (2–5.2)	0.004
Rebleeding, *n* (%)	2 (3.57)	6 (15.78)	0.058
Repeat endoscopy	3 (5.35)	6 (15.78)	0.092
Mortality, *n* (%)	1 (1.8)	11 (28.9)	<0.001
NPAR	21.8 (18.2–25.1)	30.5 (25.1–35.2)	<0.001
NLR	3.24 (1.97–5.19)	4.47 (2.96–5.96)	0.034
PLR	139.40 (97.53–173.70)	169.75 (118–194.85)	0.089
SII	818.19 (549.10–1346)	1004 (562.80–2015)	0.343

Data are presented as mean ± standard deviation or median (interquartile range, IQR), as appropriate. Categorical variables are expressed as number (percentage). NPAR: neutrophil percentage-to-albumin ratio; NLR: neutrophil-to-lymphocyte ratio; PLR: platelet-to-lymphocyte ratio; SII: systemic immune-inflammation index.

**Table 3:** Comparison of clinical and laboratory parameters according to Rockall score.

Parameter	Rockall < 5 (*n* = 43)	Rockall ≥ 5 (*n* = 51)	*p*-Value
Age, years	51 (33–65)	77 (65–83)	<0.001
Hemoglobin, g/dL	10.2 ± 2.6	8.2 ± 2.4	0.001
Platelet count, ×10^9^/L	262 (203–317)	254 (206–340)	0.943
White blood cell count, ×10^9^/L	10.1 ± 3.9	9.2 ± 3.4	0.255
Neutrophil count, ×10^9^/L	7.4 ± 3.7	6.9 ± 3.2	0.593
Neutrophil percentage, %	71.0 ± 12	73.0 ± 11	0.337
Albumin, g/dL	3.2 ± 0.4	2.7 ± 0.5	<0.001
Urea, mg/dL	65 (53–78)	81 (49–130)	0.048
Creatinine, mg/dL	0.8 (0.5–1.2)	1.4 (0.4–8.6)	<0.001
Hospitalization length (day)	2 (1–3)	3 (2–5)	0.001
Erythrocyte suspension transfusions, units	1 (0–3)	3 (2–5)	0.001
Rebleeding, *n* (%)	0 (0)	8 (15.7)	0.007
Repeat endoscopy	0 (0)	9 (17.6)	0.004
Mortality, *n* (%)	0 (0)	12 (23.5)	0.001
NPAR	21.9 (19.4–25.7)	27.4 (21.8–33)	<0.001
NLR	3.39 (2.03–5.37)	3.88 (2.36–5.48)	0.311
PLR	137.3 (91.9–189.9)	160.1 (118.6–192)	0.260
SII	817.9 (562.9–1366.8)	903 (544.4–1931.5)	0.663

Data are presented as mean ± standard deviation or median (interquartile range, IQR), as appropriate. Categorical variables are expressed as number (percentage). NPAR: neutrophil percentage-to-albumin ratio; NLR: neutrophil-to-lymphocyte ratio; PLR: platelet-to-lymphocyte ratio; SII: systemic immune-inflammation index.

**Table 4:** ROC analysis of NPAR for predicting AIMS65 score ≥ 2, Rockall score ≥ 5, and mortality.

Outcome	Cut-Off	AUC	95% CI	Sensitivity (%)	Specificity (%)	*p*-Value
AIMS65 ≥ 2	23.5	0.843	0.763–0.923	86.8	66.1	<0.001
Rockall ≥ 5	21.2	0.714	0.610–0.817	82.4	45.0	<0.001
Mortality	27.4	0.900	0.832–0.969	91.7	75.6	<0.001

AUC: area under the curve; CI: confidence interval.

**Table 5:** Correlation analysis of NPAR with clinical parameters.

Variable	r	*p*-Value
Hospitalization length (day)	0.636	<0.001
Erythrocyte suspension transfusions, units	0.559	<0.001
Rockall score	0.491	<0.001
AIMS65 score	0.660	<0.001

r: Spearman correlation coefficient.

**Table 6:** Bivariable logistic regression analysis for predicting in-hospital mortality.

Variable	OR	95% CI	*p*-Value
Age	1.049	0.986–1.116	0.128
NPAR	1.162	1.065–1.268	0.001

OR: odds ratio; CI: confidence interval.

**Table 7:** Diagnostic performance of NPAR for predicting mortality.

Outcome	Cut-Off	Odds Ratio	95% CI	PPV (%)	NPV (%)	*p*-Value
Mortality	27.4	31.9	3.88–102.59	34.4	98.4	<0.001

AUC: area under the curve; CI: confidence interval; PPV: positive predictive value; NPV: negative predictive value.

## Data Availability

The data presented in this study are available from the corresponding author upon reasonable request.
